# Hfq binding changes the structure of *Escherichia coli* small noncoding RNAs OxyS and RprA, which are involved in the riboregulation of *rpoS*

**DOI:** 10.1261/rna.034595.112

**Published:** 2013-08

**Authors:** Charlotte A. Henderson, Helen A. Vincent, Alessandra Casamento, Carlanne M. Stone, Jack O. Phillips, Peter D. Cary, Frank Sobott, Darren M. Gowers, James E. N. Taylor, Anastasia J. Callaghan

**Affiliations:** 1 Biophysics Laboratories, School of Biological Sciences, Institute of Biomedical and Biomolecular Sciences, University of Portsmouth, Portsmouth, PO1 2DT, United Kingdom; 2 Biochemistry Department, University of Oxford, Oxford, OX1 3QU, United Kingdom

**Keywords:** Hfq, sRNA, RprA, OxyS, *rpoS*

## Abstract

OxyS and RprA are two small noncoding RNAs (sRNAs) that modulate the expression of *rpoS,* encoding an alternative sigma factor that activates transcription of multiple *Escherichia coli* stress-response genes. While RprA activates *rpoS* for translation, OxyS down-regulates the transcript. Crucially, the RNA binding protein Hfq is required for both sRNAs to function, although the specific role played by Hfq remains unclear. We have investigated RprA and OxyS interactions with Hfq using biochemical and biophysical approaches. In particular, we have obtained the molecular envelopes of the Hfq–sRNA complexes using small-angle scattering methods, which reveal key molecular details. These data indicate that Hfq does not substantially change shape upon complex formation, whereas the sRNAs do. We link the impact of Hfq binding, and the sRNA structural changes induced, to transcript stability with respect to RNase E degradation. In light of these findings, we discuss the role of Hfq in the opposing regulatory functions played by RprA and OxyS in *rpoS* regulation.

## Abstract (precis)

Many small noncoding RNAs in *E. coli* require the RNA binding protein Hfq to exert their regulatory function. However, the role(s) played by Hfq remain unclear. This manuscript uses a variety of biochemical and biophysical approaches to characterize the role of Hfq in the opposing functions of two small RNAs.

## INTRODUCTION

RpoS is a σ factor (σ^s^) of stationary growth phase in *Escherichia coli* that functions as a master regulator, activating a plethora of genes involved in general stress response (for review, see Repoila et al. 2003). The *rpoS* transcript is intrinsically repressed due to extensive secondary structure in its 5′ untranslated region (UTR) that sequesters the Shine-Dalgarno site (SD) and thus impedes translation (Brown and Elliott 1997; Majdalani et al. 1998, 2002). Four small noncoding RNAs (sRNAs) regulate the translation of *rpoS,* namely, DsrA, RprA, ArcZ, and OxyS, each of which is expressed under a different stress condition. The first three sRNAs act to positively regulate the translation of *rpoS* under conditions of low temperature stress and osmotic shock and in response to aerobic/anaerobic growth conditions, respectively (Sledjeski et al. 1996; Zhang et al. 1998; Majdalani et al. 2001; Mandin and Gottesman 2010; Soper et al. 2010). Each sRNA functions by binding to the *rpoS* 5′ leader sequence, thereby opening up the inhibitory structure and allowing access to the SD site (Lease et al. 1998; Majdalani et al. 1998, 2002). Conversely, the fourth sRNA, OxyS, negatively regulates *rpoS* under conditions of oxidative stress (Altuvia et al. 1997). It is proposed to function through pairing to *rpoS*, such that translation is prevented (Zhang et al. 2002). This negative regulation is believed to serve in complex regulation that prevents redundancy in responses to stress (Altuvia et al. 1997). Crucially, the efficient regulation of the *rpoS* transcript by the sRNAs requires the RNA binding protein Hfq (Zhang et al. 1998; Majdalani et al. 2001, 2002; Sledjeski et al. 2001; Soper et al. 2010).

The *E. coli* Hfq protein has an N-terminal central core region composed of six identical subunits in a toroid conformation with long flexible C-terminal tail regions extending outward (Schumacher et al. 2002; Sauter et al. 2003; Beich-Frandsen et al. 2011a,b; Vincent et al. 2012a). The Hfq core has two distinct faces; these proximal and distal faces have both been shown to be involved in RNA binding. In particular, U-rich RNA sequences, often found in sRNAs, have been shown to interact with the proximal face of Hfq, while A-rich sequences, or A-R-N repeats (where R is a purine nucleotide and N is any nucleotide), usually found in mRNAs but also present in some sRNAs, have been shown to interact with the distal face (Schumacher et al. 2002; Mikulecky et al. 2004; Updegrove et al. 2008; Link et al. 2009; Sauer and Weichenrieder 2011; Updegrove and Wartell 2011; Wang et al. 2011). Recently, sRNA binding to the lateral surface of the Hfq core has also been identified (Updegrove and Wartell 2011; Sauer et al. 2012). However, there is debate over the role of the flexible C-terminal tails of Hfq in RNA binding. Some findings suggest that the Hfq core region alone is sufficient for riboregulatory effects, while other studies indicate that the flexible C-terminal tails may be involved in interacting with mRNAs (Vecerek et al. 2008; Olsen et al. 2010).

One of the key functions of Hfq in sRNA-mediated post-transcriptional gene regulation is to facilitate the pairing between the sRNAs and their target mRNAs. This is proposed to occur either by Hfq acting as a platform upon which both RNAs bind simultaneously, bringing the two RNA molecules into close proximity to increase their likelihood of pairing (Soper et al. 2011), or by Hfq altering the structure of one or both RNAs to expose the partner RNA interaction sites (Zhang et al. 2002; Soper et al. 2011). In addition to facilitating sRNA–mRNA pairing, Hfq can affect the overall stability of these RNAs, which is largely due to Hfq sharing the same binding/cleavage site preferences for AU-rich regions, as the major degradative endoribonuclease, RNase E (Mackie 1998; Kaberdin et al. 2000; Vytvytska et al. 2000; Zhang et al. 2002; Brescia et al. 2003). If Hfq is bound to the RNA, the protein can provide steric protection against RNase E degradation (Melefors and von Gabain 1988; Folichon et al. 2003; Moll et al. 2003). However, the binding of RNA to Hfq can also increase its susceptibility to cleavage in some cases. This is caused either by Hfq changing the structure of the RNA, leading to the exposure of novel RNase cleavage sites (Zhang et al. 2002), or by targeting specific mRNAs for degradation through the formation of specialized ribonucleoprotein complexes, comprising Hfq, RNase E, and an sRNA (Morita et al. 2005; Bandyra et al. 2012). This complex is believed to assemble on the C-terminal region (CTR) of RNase E (amino acids 530–1061) and acts to specifically guide RNase E on to the target mRNA for endonucleolytic degradation. Although many roles have been attributed to Hfq, its exact mechanism of action within the context of these differing sRNA-mediated post-transcriptional gene regulatory effects remains unclear.

**FIGURE 1.**
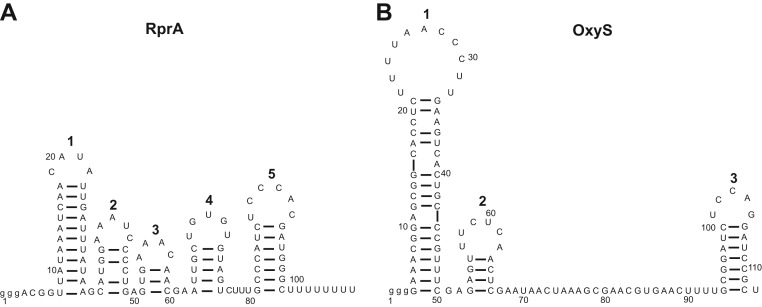
Structures of RprA and OxyS. (*A*) Predicted secondary structure of RprA determined using Mfold (Zuker 2003). Five unequally sized stem–loops are predicted. (*B*) Experimentally verified secondary structure of OxyS (Zhang et al. 2002), which is in close agreement with the Mfold prediction. In both *A* and *B*, the stem–loops are numbered.

This study focuses on two sRNAs that differentially regulate *rpoS,* namely, RprA and OxyS (Fig. 1A,B). These sRNAs are of interest for assessing if Hfq works differently for an sRNA that positively regulates an mRNA target (RprA) compared with one that negatively regulates its mRNA target (OxyS). Both sRNAs have already been seen to form complexes with Hfq (Updegrove et al. 2008; Olejniczak 2011; Updegrove and Wartell 2011), with RprA shown to have interactions with both the proximal and distal face of Hfq, while OxyS has interactions with the proximal, distal, and lateral surfaces (Updegrove and Wartell 2011). The Hfq interaction with OxyS has also been shown to result in a structural change in the sRNA (Zhang et al. 2002), but for RprA, it remains unknown whether this occurs. We further characterize these interactions, initially by verifying that both RprA and OxyS can accommodate Hfq and subsequently by providing details on the complex stoichiometries, affinities, and low-resolution structures. We then structurally model both sRNA:Hfq complexes and demonstrate that Hfq can alter the structure of both sRNAs and provide ab initio models of these changes. Finally, we assess how the interaction with Hfq affects the susceptibility of OxyS and RprA to RNase E attack.

## RESULTS

### Binding affinities of sRNA:Hfq complexes

Hfq interacts promiscuously with many RNAs containing an AU-rich sequence adjacent to a stem–loop (Brescia et al. 2003; Moll et al. 2003). This lack of binding specificity can result in multiple Hfq hexamers binding to a single RNA, as is the case for the sRNAs RprA and OxyS. These have both been previously shown to form two discrete complexes with Hfq, namely, complexes I and II (Updegrove et al. 2008; Olejniczak 2011; Updegrove and Wartell 2011), with complex I suggested to consist of a 1:1 sRNA:Hfq hexamer stoichiometric ratio (Updegrove et al. 2011), while the sRNA:Hfq hexamer stoichiometric ratio within complex II remains unknown.

**FIGURE 2.**
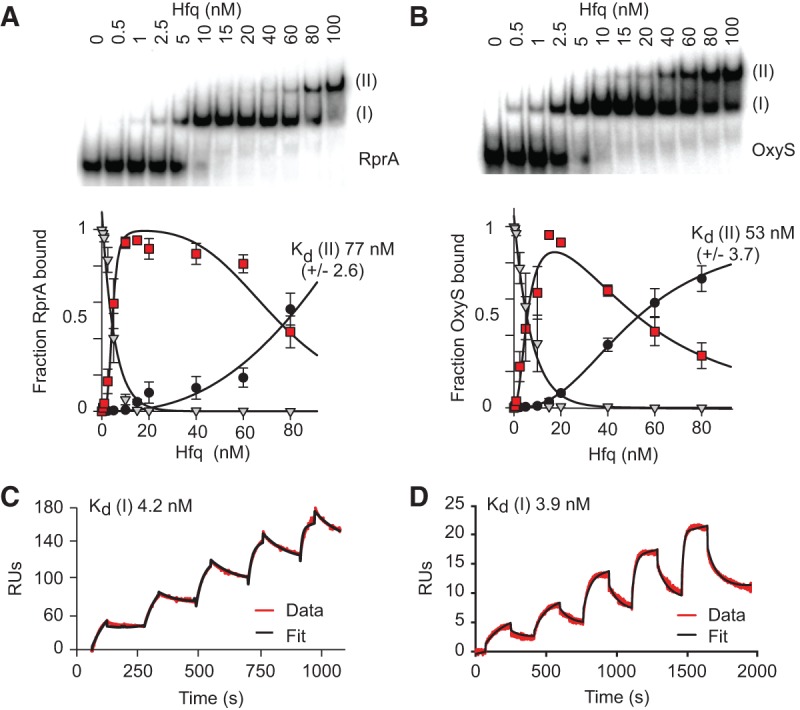
Affinity of sRNA:Hfq complexes. ^32^P RprA (5 nM; *A*) or ^32^P OxyS (5 nM; *B*) were incubated at 25°C with increasing concentrations of Hfq hexamer (0–100 nM). Samples were analyzed by native PAGE at room temperature. Complexes I and II and free sRNA are indicated to *left* of the gel. On the corresponding graphical representation of the EMSA data, gray triangles represent free RNA, red squares represent complex I, and black circles represent complex II. RprA:Hfq and OxyS:Hfq complexes were fit using a two-site partition model (Lease and Woodson 2004). The complex II *K*_d_s are shown on the graphs. (*C*) SPR of RprA at 3.125 nM, 6.25 nM, 12.5 nM, 25 nM, and 50 nM flowed over Hfq immobilized on a CM5 chip. Binding is in arbitrary response units (RUs). The red line represents the data, and the black line is the fit of the data to a 1:1 binding model, producing a χ^2^ value of 3.52 RU^2^. The complex I *K*_d_ is shown on the sensorgram. (*D*) SPR of OxyS at 1.56 nM, 3.125 nM, 6.25 nM, 12.5 nM, and 25 nM flowed over Hfq immobilized on a CM5 chip. The red line represents the data, and the black line the fit of the data to a 1:1 binding model, producing a χ^2^ of 0.338 RU^2^. Only 1:1 complexes would be formed under these conditions. The complex I *K*_d_ is shown on the sensorgram.

To determine the dissociation constants (*K*_d_) for complexes I and II for RprA and OxyS, electrophoretic mobility shift assay (EMSA) experiments were undertaken (Fig. 2A,B) and the binding data fit to a two-site partition model (Lease and Woodson 2004). For RprA, the data gave a *K*_d_ of 4.7 nM for complex I and a *K*_d_ of 77 nM for complex II. For OxyS, the data gave a *K*_d_ of 5.6 nM for complex I and a *K*_d_ of 53 nM for complex II. While these values are similar to the dissociation constants previously determined for complex I of both RprA:Hfq and OxyS:Hfq and complex II of RprA:Hfq (Updegrove et al. 2008; Olejniczak 2011; Updegrove and Wartell 2011), we note that the sRNAs used here incorporated 3′pCp labeling, which might block Hfq recognition of the 3′OH (Sauer and Weichenrieder 2011). Additionally, the concentrations of sRNA used (5 nM) were near the *K*_d_ values for complex I for both RNAs. When the initial rise in complex I concentration (between 0 and 20 nM Hfq concentration) was fit to a quadratic function, a *K*_d_ of 4.3 nM was found for RprA:Hfq and 8.0 nM for OxyS:Hfq (data not shown), values similar to those identified using the partition model above. Therefore, to accurately determine the *K*_d_ of complex I, surface plasmon resonance (SPR) was used to analyze nonlabeled RprA and OxyS binding to immobilized Hfq. Based on the EMSA data, a concentration range of 0–50 nM RprA and 0–25 nM OxyS was used such that predominantly only complex I would be formed. The data fitted a 1:1 binding model (Fig. 2C,D) and gave a *K*_d_ of 4.2 nM for RprA:Hfq complex I and 3.9 nM for OxyS:Hfq complex I. The similar *K*_d_s determined for the nonlabeled sRNAs compared with the *K*_d_s estimated from EMSA analysis suggest that while 3-OH end recognition may be important for binding to Hfq, it is not a critical determinant impacting binding affinity for these sRNAs. Additionally, we note that the OxyS sequence used in this study had its 3′ poly(U) tail removed to allow comparison with previous OxyS studies (Altuvia et al. 1997, 1998; Olejniczak 2011; Updegrove and Wartell 2011). While recent studies indicate the importance of the 3′ poly(U) tail in Hfq binding (Otaka et al. 2011; Sauer and Weichenrieder 2011; Ishikawa et al. 2012), tests to compare OxyS ± poly(U) tail binding to Hfq indicated no difference (data not shown). Hence, our studies proceeded using the currently studied form of OxyS, lacking the 3′ poly(U) tail, to allow for consistency with earlier work.

### Stoichiometry of sRNA:Hfq complexes

**FIGURE 3.**
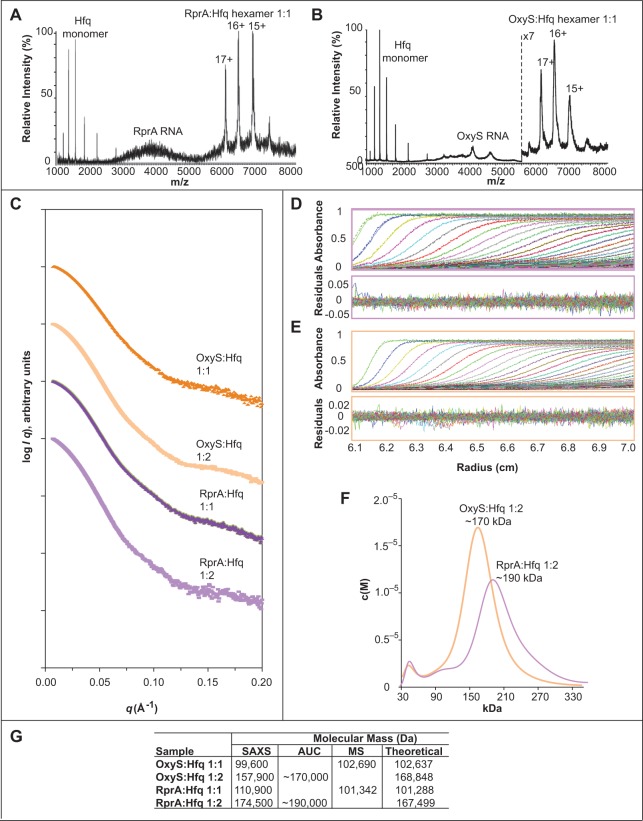
Stoichiometry of sRNA:Hfq complexes. Nondenaturing MS of 5 µM RprA with 5 µM Hfq hexamer (*A*) and 2.36 µM OxyS with 2.36 µM Hfq hexamer (*B*). The OxyS:Hfq hexamer 1:1 has ×7 zoom (after the vertical dotted line). (*C*) SAXS scattering curves of OxyS:Hfq hexamer 1:1 (dark orange) and 1:2 (light orange) and RprA:Hfq hexamer 1:1 (dark purple) and 1:2 (light purple). (*D*) AUC absorbance scans of 1:2 RprA:Hfq hexamer complex with corresponding residuals showing the goodness of fit to the data. (*E*) As for *D* but for 1:2 OxyS:Hfq hexamer complex. (*F*) AUC c(M) plot fitted using SEDFIT (Schuck 2000) of 1:2 RprA:Hfq hexamer complex (light purple) and 1:2 OxyS:Hfq hexamer complex (light orange). (*G*) Table showing the experimental molecular mass values for the samples calculated from MS, SAXS, and AUC data analysis. The theoretical values are shown for comparison.

To evaluate the compositional stoichiometries of complexes I and II, nondenaturing mass spectrometry (MS) and analytical ultracentrifugation (AUC) were performed. Complex I has been suggested to consist of a 1:1 ratio of sRNA:Hfq hexamer from the data above and studies by the Wartell group (Updegrove et al. 2011). To generate complex I an equimolar ratio of sRNA to Hfq hexamer were mixed. The stoichiometry of complex I was investigated by MS, selecting parameters to preserve intact noncovalent interactions (Sobott et al. 2005, 2010). The results showed a peak series containing predominantly three charge states (+15 to +17) (Fig. 3A,B,G). For RprA:Hfq, this series corresponds to a molecular mass of 101,342 Da, while for OxyS:Hfq, this corresponds to a molecular mass of 102,690 Da. These experimental complex masses were very similar to the theoretical values of the 1:1 stoichiometries (101,288 Da for RprA:Hfq hexamer; 102,637 Da for OxyS:Hfq hexamer), indicating that complex I consists of one molecule of RNA and one Hfq hexamer. Similarly, small-angle X-ray scattering (SAXS) analysis also confirmed the 1:1 sRNA:Hfq stoichiometries for the equimolar samples of RprA or OxyS with Hfq (Fig. 3C,G). Additionally, following SAXS analysis, the samples used were analyzed by native polyacrylamide gel electrophoresis (PAGE), and the same mobility shift was identified as that seen for complex I by EMSA (data not shown).

The stoichiometry of complex II was subsequently investigated by AUC and SAXS to see whether it represents a 1:2 sRNA:Hfq hexamer complex, as the two-site binding model used to fit the EMSA data above suggests at least two Hfq sites are available on the sRNAs for binding. To generate complex II, each sRNA was mixed with Hfq hexamer at a molar ratio of 1:2. For RprA:Hfq hexamer, experimental molecular masses of ∼190 kDa and 174.5 kDa were identified by AUC and SAXS analysis, respectively (Fig. 3C–G). In addition, for OxyS:Hfq, the experimental molecular masses of ∼170 kDa and 157.9 kDa were identified by AUC and SAXS analysis, respectively (Fig. 3C–G). These are in close agreement with the theoretical molecular mass values for 1:2 sRNA:Hfq complexes (167.5 kDa for 1:2 RprA:Hfq hexamer; 169 kDa for 1:2 OxyS:Hfq hexamer).

Overall, this stoichiometric assessment agrees with our binding data and earlier suggestions that complex I is a 1:1 sRNA:Hfq hexamer ratio (Updegrove et al. 2011) and that complex II is a 1:2 ratio of sRNA:Hfq hexamer for both RprA and OxyS.

### Hfq changes the structure of OxyS and RprA

**FIGURE 4.**
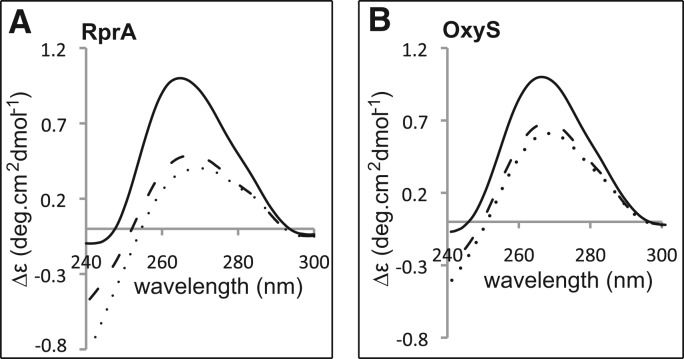
RprA and OxyS structural changes upon Hfq addition. (*A*) CD molar ellipticity of 1 µM RprA (solid line), 1 µM RprA with 1 µM Hfq hexamer (i.e., 1:1 RprA:Hfq hexamer; dashed line), and 1 µM RprA with 2 µM Hfq hexamer (i.e., 1:2 RprA:Hfq hexamer; dotted line). (*B*) CD molar ellipticity of 1 µM OxyS (solid line), 1 µM OxyS with 1 µM Hfq hexamer (i.e., 1:1 OxyS:Hfq; dashed line), and 1 µM OxyS with 2 µM Hfq hexamer (i.e., 1:2 OxyS:Hfq hexamer; dotted line).

The interaction of Hfq with OxyS is known to induce a structural change in the sRNA (Zhang et al. 2002). However, it is currently not known whether Hfq causes any structural changes to occur in RprA. To determine whether the structure of RprA is altered upon Hfq interaction, both OxyS and RprA were assessed by circular dichroism (CD) (Fig. 4). The RNAs were initially measured individually and then with addition of Hfq to a stoichiometric ratio of 1:1 and 1:2 sRNA:Hfq hexamer. The protein contribution within the wavelength range of 240–350 nm of the CD spectrum was subtracted, but due to the small number of aromatic residues within Hfq, the contribution was negligible. This means the peaks at 240–350 nm are dominated by the contribution from the RNA (Daly et al. 1990; Tan and Frankel 1992; Aparicio et al. 2003; Vincent et al. 2012b; Henderson et al. 2013).

For RprA, the addition of Hfq hexamer to a 1:1 stoichiometric ratio reduced the ellipticity at 265 nm by 55% (1.00 Δε to 0.46 Δε) (Fig. 4A). There was also a loss in peak width in the 250-nm region and a 2-nm shift in the maximum from 265 nm to 267 nm. These observations are indicative of loss of double-stranded structure in the sRNA, as determined by the assessment of the spectra profiles, which followed the same spectra patterns to sRNAs that had been heated to melt their double-stranded structures (data not shown; Vincent et al. 2012b). Addition of Hfq hexamer to a 1:2 RprA:Hfq stoichiometric ratio, showed only a small further decrease in ellipticity (0.46 Δε to 0.37 Δε) (Fig. 4A), which may be more indicative of a slight chromophore rearrangement upon Hfq binding, rather than a significant structural change (Aparicio et al. 2003; Vincent et al. 2012b; Henderson et al. 2013). For OxyS, the addition of Hfq hexamer to a 1:1 stoichiometric ratio reduced the ellipticity at 265 nm by 35% (1.00 Δε to 0.65 Δε) (Fig. 4B). Similarly to RprA, there was also a loss in peak width in the 250-nm region and a 2-nm shift in ellipticity maxima from 265 nm to 267 nm. Again these observations are indicative of loss of double-stranded structure and are consistent with the previous findings that show Hfq changes the structure of OxyS (Zhang et al. 2002). The greater ellipticity change of RprA upon Hfq addition in comparison to that observed for OxyS (55% vs. 35%) suggests a more significant structural rearrangement occurs in RprA upon Hfq binding, a finding not entirely unexpected as programs that predict RNA secondary structure (Mfold) (Zuker 2003) suggest that RprA contains a greater proportion of secondary structure compared with OxyS (Fig. 1A,B). Furthermore, like RprA, the addition of Hfq hexamer to a 1:2 OxyS:Hfq stoichiometric ratio showed only a small further decrease in ellipticity at 265 nm (0.65 Δε to 0.60 Δε), suggestive more of a binding rearrangement rather than further structural change (Aparicio et al. 2003; Vincent et al. 2012b; Henderson et al. 2013).

Overall these data suggest that the first Hfq binding event (to form complex I) induces the greatest structural rearrangement to both sRNAs, while the second Hfq binding (to form complex II) does not remodel the sRNA structure as extensively.

### Solution structures of OxyS and RprA in isolation and in 1:1 complexes with Hfq

**FIGURE 5.**
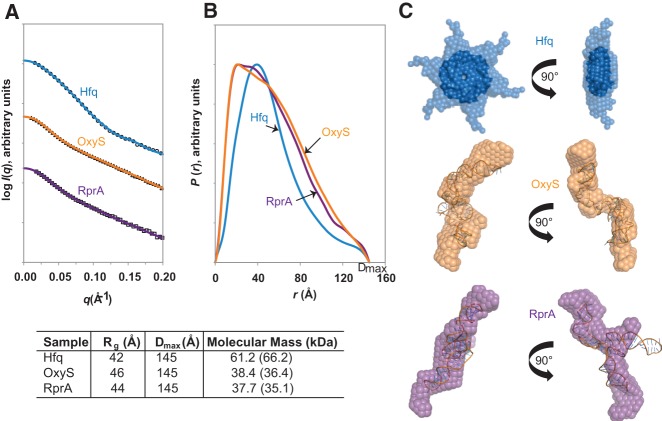
SAXS data of Hfq, OxyS, and RprA. (*A*) SAXS data of Hfq hexamer (circles), OxyS (triangles), and RprA (squares). The solid lines (Hfq, blue; OxyS, orange; and RprA, purple) represent the back-transformed distance distribution functions, *P*(*r*), that are shown in *B*. *R*_g_s and *D*_max_ values, together with the molecular mass values calculated for the SAXS data and the theoretical values in parentheses, are given in the table *beneath*. (*C*) Ab initio models produced from *B*. Hfq (blue), OxyS (orange), and RprA (purple). The crystal structure of *E. coli* Hfq (PDB 1HK9), displayed as dark blue spacefill, was docked into the ab initio model of Hfq using SUPCOMB (Kozin et al. 2001). The missing density of the crystal structure that is present in the ab initio model is attributed to the C-terminal tails of Hfq, which are lacking in the crystal structure. The predicted three-dimensional structures of OxyS and RprA, produced by iFold RNA (Sharma et al. 2008) and displayed in stick representation, were docked into the ab initio models for each sRNA, using SUPCOMB (Kozin and Svergun 2001).

To further assess how the structures of the sRNAs are changed upon addition of Hfq, SAXS and small-angle neutron scattering (SANS) experiments were performed. First, SAXS data were collected for a tRNA control, and iFold RNA software (Sharma et al. 2008) was used to generate a tRNA model. The iFold tRNA model was superimposed onto the SAXS tRNA envelope and corresponding crystal structure (Protein Data Bank [PDB] 1EHZ) (Shi and Moore 2000). The superimposition between all three was observed to be good (data not shown), indicating confidence in iFold RNA structure predictions. This demonstrated the validity of the approach of comparing predicted iFold RNA structures to molecular envelopes from SAXS analysis in terms of providing an appropriate gauge of RNA structure and size. SAXS data were then collected for RprA and OxyS, and data analysis identified the radius of gyration (*R*_g_) as 44 and 46 Å, respectively, and maximum dimension (*D*_max_) values for both to be 145 Å (Fig. 5). The ab initio models generated indicated the sRNAs to both be rod shaped with distinct nodules. iFold RNA software (Sharma et al. 2008) was used to predict the structures of the two sRNAs, and these were docked into their respective molecular envelopes derived by SAXS (Fig. 5C). Considering the results for OxyS and RprA, the data indicated agreement between the predicted iFold RNA models and the molecular envelopes from SAXS (Fig. 5C), with the nodules of the ab initio models indicative of the presence of loops or folds within each sRNA. Additionally, the calculation of the molecular masses from the SAXS data confirmed both sRNAs to be monomeric in solution. Experimentally determined values of 38.4 kDa for OxyS and 37.7 kDa for RprA compared favorably to the theoretical values of 36.4 kDa and 35.1 kDa for OxyS and RprA, respectively. SAXS data were also collected for uncomplexed Hfq hexamer, confirming previous observations of it being hexameric in solution (experimentally determined molecular mass of 61.2 kDa, compared with the theoretical value of 66.2 kDa) (Beich-Frandsen et al. 2011a; Vincent et al. 2012a). The ab initio model generated for Hfq formed a symmetrical star-shaped conformation, consistent with the low-resolution structures previously reported (Beich-Frandsen et al. 2011a; Vincent et al. 2012a). Minor variations in *R*_g_ and *D*_max_ values compared with earlier published reports are attributed to the low-salt Hfq buffer conditions that were used to optimize the Hfq:sRNA interactions, compared with the high-salt conditions used in previous studies of Hfq in isolation. We suggest that under these low-salt conditions, Hfq displays an increased flexibility, potentially within the C-terminal tail regions.

**FIGURE 6.**
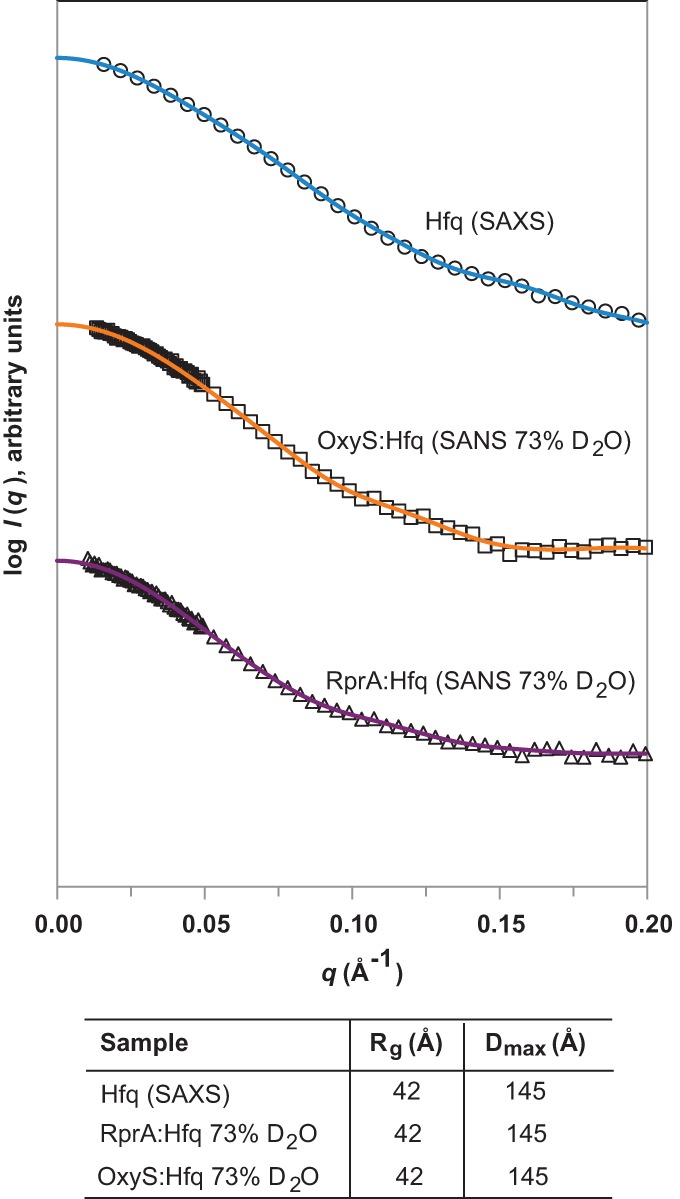
Comparison of Hfq SAXS data and 73% SANS data of 1:1 sRNA:Hfq complexes. SAXS data of free Hfq hexamer (blue line and circles). SANS data measured in 73% D_2_O of Hfq in 1:1 complex with OxyS (orange line with squares) or RprA (purple line with triangles). The corresponding *R*_g_ and *D*_max_ values are given in the table *beneath*.

**FIGURE 7.**
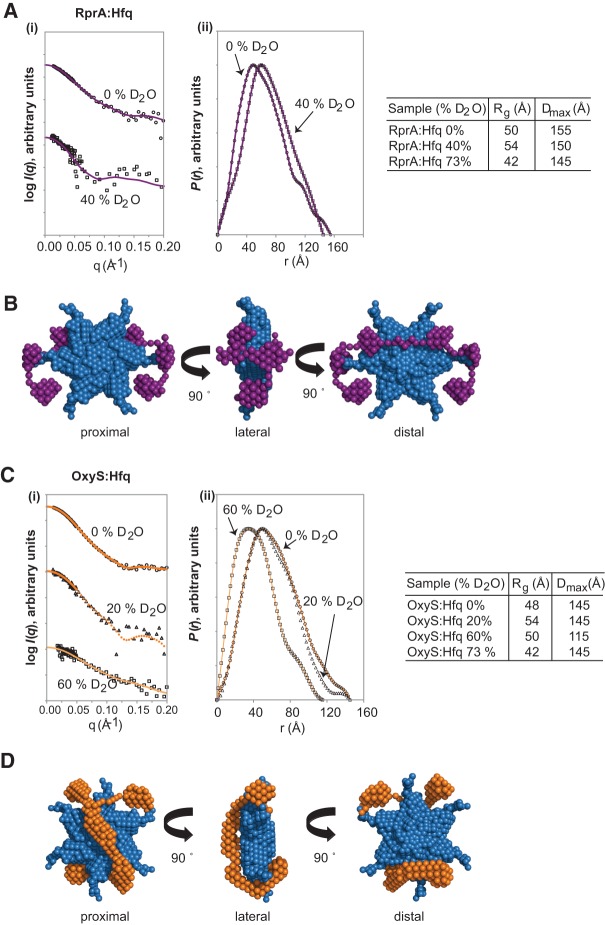
SANS contrast variation data and MONSA ab initio model of Hfq in complex with RprA or OxyS. (*Ai*) SANS data of 1:1 RprA:Hfq hexamer complex in 0% (circles) and 40% (squares) D_2_O. Fits to 0 and 40% SANS data had χ values of 1.0 and 1.4, respectively. (*Aii*) Corresponding *P*(*r*) plots of the data in *Ai* along with a table giving the *R*_g_ and *D*_max_ values. (*B*) MONSA ab initio model of the 1:1 RprA:Hfq hexamer complex. Hfq (blue spheres) and RprA (purple spheres). The protein phase (Hfq) was first modeled with DAMMIF with P6 symmetry imposed. Theoretical volumes and *R*_g_ values determined from Guinier analyses were used as further constraints during the modeling process, as was the overall *R*_g_ for the complex. The Hfq-face labels of proximal, lateral, and distal are suggestions based on the conclusions of earlier published data discussed within the text. (*Ci*) SANS scattering data of 1:1 OxyS:Hfq hexamer complex in 0% (circles), 20% (triangles), and 60% (squares) D_2_O. Fits to 0%, 20%, and 60% SANS data had χ values of 1.6, 3.0, and 5.0, respectively. (*Cii*) Corresponding *P*(*r*) plots of the data in *Ci* along with a table giving the *R*_g_ and *D*_max_ values. (*D*) MONSA ab initio model of the 1:1 OxyS:Hfq hexamer complex. Hfq (blue spheres) and OxyS (orange spheres). Modeling was performed as in *B*.

Characterization of the 1:1 RprA:Hfq hexamer and 1:1 OxyS:Hfq hexamer complexes was undertaken by SANS experiments. The 1:1 complexes were chosen for analysis as they were identified as likely to be the most functionally important, since CD analysis indicated the most significant sRNA structural changes occurred at this sRNA:Hfq hexamer ratio. SANS was performed to assess the low-resolution structure of the complexes and determine the conformation of the sRNAs within the complex. For these experiments, a series of different D_2_O:H_2_O ratios were used collectively to create contrast variation, which enabled the location of the sRNA and Hfq hexamer within the complex to be individually identified. For example, in 0% D_2_O the scattering contains the full contribution from both the RNA and the protein, whereas in ∼40% D_2_O, the scattering represents the scattering due to RNA alone, and in 73% D_2_O, the scattering shows only the contribution from the protein. Therefore by comparing the shape, *R*_g_, and *D*_max_ of the 73% D_2_O scattering curves for sRNA:Hfq hexamer complexes (showing the Hfq contribution only) with the SAXS scattering curve of the uncomplexed Hfq hexamer, it can be seen that the scattering data are not significantly different (Fig. 6). This suggests that the Hfq hexamer does not significantly alter shape upon binding to either RprA or OxyS (Fig. 6). Ab initio modeling of the SANS data for 0%, 20%, 40%, and 60% D_2_O concentrations, while maintaining the known Hfq conformation (determined under low-salt conditions), provided details on the conformation of the sRNAs when in complex with the Hfq hexamer. Theoretical volumes and *R*_g_ values determined from Guinier analyses were used as constraints during the modeling process. These indicated that the overall solution structures of the sRNAs in their Hfq-bound state (Fig. 7) were significantly altered from the solution structure of the sRNAs individually (Fig. 5). This confirmed that the Hfq hexamer affects the structure of RprA and OxyS, which is in agreement with our CD analysis; as for both sRNAs, binding to the Hfq hexamer caused them to form more extended conformations.

Furthermore, the overall ab initio models of each sRNA:Hfq complex showed that both RprA and OxyS predominantly contacted one side of the Hfq hexamer, although limited interactions with the opposite and lateral faces of Hfq were also seen. While the resolution of scattering techniques cannot distinguish between the proximal and distal face of Hfq, based on previous studies, it is possible that RprA is predominantly binding to the distal face whereas OxyS is predominantly binding to the proximal face (Zhang et al. 2002; Updegrove et al. 2008; Updegrove and Wartell 2011). Earlier work also suggests that the mRNA target of RprA and OxyS, namely, *rpoS*, would bind predominantly to the distal face of Hfq, although an interaction with the lateral surface has been noted (Updegrove and Wartell 2011). Interestingly, in both ab initio models, there are regions of the distal and lateral surfaces that are exposed and could therefore accommodate *rpoS* binding. Additionally, the CTRs of Hfq in both ab initio models remain fairly unoccupied. While there remains debate over the role of the Hfq C-terminal tails in RNA binding, previous findings suggest that they are important with respect to *rpoS* binding (Vecerek et al. 2008; Updegrove and Wartell 2011). The free C-terminal tails in the sRNA:Hfq ab initio models determined here would support this possibility.

### Hfq impact on RNA stability

**FIGURE 8.**
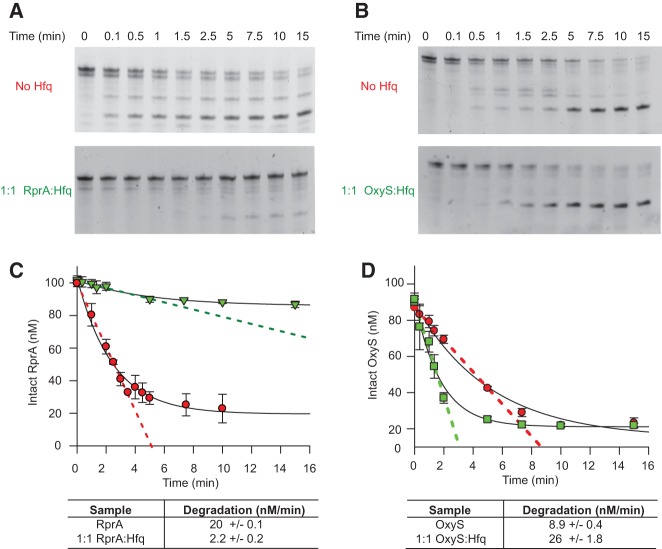
Hfq protects RprA but enhances OxyS degradation by RNase E. (*A*) RprA (100 nM) and RNase E (100 nM) were incubated over 15 min and analyzed by denaturing PAGE (*top* gel) and then with the addition of 100 nM Hfq hexamer to form a 1:1 RprA:Hfq hexamer complex (*bottom* gel). The gels were stained with SYBR Gold and visualized under UV transilluminescence. (*B*) As for *A*, but with OxyS instead of RprA. (*C*) Graphical representation of the RprA degradation data in *A*. Red circles represent RprA degradation upon addition of 100 nM RNase E, and green triangles represent RprA degradation upon addition of 100 nM RNase E plus 100 nM Hfq hexamer. The SEM was based on three experimental repeats. The initial rate of degradation of RprA (0–3 min) without Hfq (red) or with 100 nM Hfq (green) are shown by the dashed lines (linear fits to the data) and given in the table *beneath*. (*D*) As for *C*, but for the OxyS data in *B*.

Hfq has already been shown to enhance the pairing of certain sRNAs with their target mRNAs, by acting as a platform upon which both sRNAs and mRNAs can bind, allowing them to come into close proximity with the consequence that they pair more easily (Soper and Woodson 2008). More specifically, for RprA the addition of Hfq has been shown to result in a 30-fold increase in RprA-*rpoS* pairing (Updegrove et al. 2008). However, for OxyS, the addition of Hfq results in very little effect on OxyS–*rpoS* pairing (Updegrove and Wartell 2011). Therefore, while Hfq has a clear role in RprA–*rpoS* pairing, its role in OxyS–*rpoS* pairing is less apparent. It is possible that with respect to OxyS, Hfq’s primary role is not in facilitating pairing to *rpoS*. Since Hfq and RNase E are known to share the same binding site preferences, Hfq may have an alternative role in the OxyS–*rpoS* interaction linked to altering RNA stability. Accordingly, we assessed the extent to which RprA and OxyS are protected from RNase E cleavage when they are in complex with Hfq. RNase E cleavage assays were performed and monitored by denaturing PAGE (Fig. 8). In vivo, sRNAs are generated as 5′ triphosphorylated RNAs. Preliminary work indicated that high concentrations of RNase E (100- to 1000-fold excess) were required to cleave the sRNAs in 5′-triphosphorylated form (data not shown). RNase E is known to preferentially cleave 5′ monophosphorylated RNA substrates (Mackie 1998), and recent studies have suggested that processing to form 5′ monophosphorylated RNAs, potentially via the action of RppH, a pyrophosphohydrolase (Deana et al. 2008), may be important for an RNase E–dependent sRNA degradation pathway (Bandyra et al. 2012). Consequently, RprA and OxyS were tested in 5′ monophosphorylated form. The degradation assays were conducted using Hfq hexamer at a 1:1 sRNA:Hfq stoichiometric ratio, since our earlier CD studies had indicated that the 1:1 complex induced the largest sRNA structural change.

For RprA, which positively regulates its target mRNA (i.e., enabling translation), sRNA degradation was significantly reduced upon Hfq binding (Fig. 8A,C). This finding is consistent with the hypothesis that Hfq is able to block the RNase E cleavage sites, due to their similar binding/cleavage preferences, and thus prevent degradation. However, our data suggest Hfq binds and causes a structural change to RprA; therefore, Hfq may not only block the RNase E cleavage site but also could sterically change the sRNA structure to remove the RNase E cleavage site altogether.

For OxyS, a negative regulator of its target mRNA, the RNase E protection effect was clearly reversed (Fig. 8B,D). OxyS degradation was increased upon Hfq binding. In this instance, since the structure of OxyS is known to change upon Hfq binding (from CD and SANS data) (Figs. 4, 7) it is possible that a more favorable RNase E cleavage site becomes exposed in the RNA, leading to enhanced RNase E cleavage. It has been proposed that Hfq could be targeting OxyS for degradation through the formation of a higher order ribonucleoprotein complex (Morita et al. 2005). However, the construct of RNase E used in these studies lacked the C-terminal scaffolding domain required for ribonucleoprotein assembly. This consequently favors the hypothesis that Hfq binding physically alters the structure of OxyS to reveal an RNase E cleavage site for the purposes of enhancing degradation of the sRNA. This suggests Hfq has a role in regulating OxyS signal removal.

## DISCUSSION

Many sRNAs require the RNA chaperone protein Hfq to fulfill their riboregulatory role, but for the majority of these RNAs, it is not known why the protein is needed. To gain a better understanding of why *E. coli* Hfq is important in sRNA-mediated communication, we compared its interactions with sRNAs that are differentially expressed in vivo and have opposing regulatory functions, using structural and biochemical methods. The sRNAs investigated were OxyS, which down-regulates *rpoS* translation in response to oxidative stress, and RprA, which up-regulates *rpoS* translation in response to osmotic shock.

We first demonstrated that two Hfq hexamers were able to bind each sRNA with low nanomolar affinity. Upon forming the 1:1 sRNA:Hfq hexamer complex (complex I), large structural rearrangements of the sRNAs occurred. This was seen by CD, SAXS, and SANS analysis. The structural changes in OxyS upon Hfq interaction have previously been mapped to the region of stem–loop 2 and the top of stem–loop 1 (Fig. 1B; Zhang et al. 2002), but for RprA, the specific structural rearrangements remain unknown. While Hfq binding was seen to induce structural changes in OxyS and RprA, it was found that Hfq itself does not undergo a significant structural change upon binding to the sRNAs.

The second Hfq binding event to form a 1:2 sRNA:Hfq hexamer complex (complex II) resulted in only small effects in terms of impact on sRNA structure changes. This could indicate complex II to be less functionally important. Although it cannot be ruled out that the second Hfq binding event could bring about a steric effect, that is not dependent upon significant structural changes and is of physiological relevance. However, considering the in vivo relevance of these sRNA:Hfq hexamer stoichiometric ratios, the number of Hfq hexamers per cell has been suggested to range from approximately 400 to 10,000 (Carmichael et al. 1975; Kajitani et al. 1994; Ali Azam et al. 1999). While some sRNAs have been identified at the level of approximately 200 copies per cell (Sahagan and Dahlberg 1979), OxyS, for example, has been identified to be present at a level of 4500 copies per cell following oxidative stress (Altuvia et al. 1997). Given these values, the fact that many different sRNAs exist within a single cell at any one time and the tight affinities observed, in general, for sRNA:Hfq interactions (Lease and Woodson 2004; Mikulecky et al. 2004; Fender et al. 2010; Olejniczak 2011), it is possible that the 1:1 RprA/OxyS:Hfq hexamer complexes are more physiologically relevant (De Lay et al. 2013). This is further supported by the in vitro data showing that significant sRNA structural changes are observed upon formation of the 1:1 sRNA:Hfq hexamer complexes. Furthermore, recent in vivo studies suggest that Hfq can, in fact, be limiting when sRNAs and their mRNA targets are transcribed at high levels (Hussein and Lim 2011). Collectively these data highlight that it is unlikely that the 1:2 RprA/OxyS:Hfq hexamer complex is of importance in vivo, and consequently, we have focused our discussion on the relevance of the 1:1 sRNA:Hfq hexamer complexes.

The interaction surfaces of Hfq involved in binding RprA and OxyS have been previously studied using a variety of methods. Specifically, Hfq mutants with residue changes on the proximal, distal, and lateral surfaces, as well as Hfq truncates lacking the C-terminal tail region have been assessed. These results, coupled with assays probing for inhibition of RprA and OxyS binding to Hfq in the presence of known distal and proximal face binders, have collectively provided insights into the sites of RprA and OxyS binding on Hfq (Updegrove et al. 2008; Olejniczak 2011; Updegrove and Wartell 2011). However, our low-resolution structural data provide the first visualization of the 1:1 sRNA:Hfq hexamer complexes.

In agreement with previous findings, the ab initio models of the sRNA:Hfq complexes suggest that both RprA and OxyS are able to contact both proximal and distal faces of Hfq. However, our data suggest that the sRNAs both show a preference for predominantly binding to one face of Hfq. The distal face of Hfq is known to bind RNA regions that are A-rich or contain A-R-N repeats (where R is a purine nucleotide and N is any nucleotide) (Link et al. 2009). Such sequence features are usually found in mRNA sequences. However, we propose that distal face binding to Hfq represents the predominant interaction seen for RprA. First, RprA has four repeats of A-R-N-N′ within its sequence, inferring capability for distal face binding to Hfq (Link et al. 2009). Second, previous data suggest that poly(A), which binds to Hfq in a ring conformation mid-way between the center and the outer edge of Hfq’s distal face, displaces RprA from Hfq (Link et al. 2009; Updegrove and Wartell 2011). Third, the mutation of K31, a residue located mid-way between the center and the outer edge of Hfq’s distal face, impacts RprA binding (Updegrove and Wartell 2011). Considering the ab initio model of the RprA:Hfq hexamer, the sRNA is seen to straddle the face of the Hfq toroid, interacting with residues mid-way between the center and outer edge. These data together indicated predominant binding of RprA to the distal face. However, it is interesting to note that mutation of F39, a residue toward the outer edge of Hfq’s proximal face, also significantly impacts binding (Updegrove and Wartell 2011), as do mutations of the lateral surface of Hfq (Sauer et al. 2012). These findings also remain in keeping with the RprA:Hfq hexamer ab initio model, which suggests that RprA wraps around the lateral surface of Hfq and is therefore capable of contacting the outer edge of the proximal face.

We propose that in contrast to RprA, OxyS binds predominantly to the proximal face of Hfq. Earlier studies have identified that the proximal face is involved in binding U-rich regions of RNA (Schumacher et al. 2002). Such U-rich regions often proceed and follow the Rho-independent terminator hairpin found at the 3′ end of sRNAs (Sauer and Weichenrieder 2011; Ishikawa et al. 2012). To be consistent with previous work, the OxyS sequence used in this study had its 3′ poly(U) tail removed (Altuvia et al. 1997, 1998; Olejniczak 2011; Updegrove and Wartell 2011). However, recent findings have highlighted the importance of this U-rich region, coupled with the presence of the 3′OH, in Hfq binding (Otaka et al. 2011; Sauer and Weichenrieder 2011; Ishikawa et al. 2012); although the role of the OxyS 3′ poly(U) tail remains to be elucidated. Nevertheless, the binding of OxyS to the proximal face is in keeping with previously published data for OxyS lacking a 3′ poly(U) tail. This earlier work showed that mutations of proximal face residues disrupted OxyS:Hfq interactions, whereas mutations of distal face residues had a much reduced effect. Additionally, the mutation of R16 on the lateral surface of Hfq was shown to significantly impact OxyS binding (Updegrove and Wartel 2011). These data are in keeping with the ab initio model of the OxyS:Hfq complex, which shows significant OxyS binding across one face of Hfq, proposed to be the proximal face, before wrapping around the lateral surface and contacting a small region of the opposite, proposed distal face, of Hfq.

The flexible CTRs of Hfq, within the OxyS/RprA:Hfq hexamer ab initio models, are seen to be fairly free of interactions with the sRNAs. However, the role of the Hfq C-terminal tails in RNA binding remains to be resolved. Previous work has suggested these regions to be dispensable for riboregulation as Hfq mutants lacking these tails were found to be functional (Olsen et al. 2010). It may therefore be considered unsurprising that the sRNAs do not significantly contact these regions within the sRNA:Hfq hexamer complex. In contrast, other work has shown that the mRNA target of RprA and OxyS, namely, *rpoS*, specifically interacts with the flexible CTRs of Hfq (Vecerek et al. 2008; Updegrove and Wartell 2011). Thus, the exposure of these regions within the sRNA:Hfq hexamer complexes would allow mRNA interactions to occur in conjunction with sRNA binding, such that a ternary complex could be formed.

Considering ternary complex formation of the sRNAs with Hfq and their common mRNA target, *rpoS*, it is known that *rpoS* contains a repetitive AAN sequence and has been shown to bind tightly to the distal face of Hfq, although an interaction with R16 on the lateral surface has also been noted (Soper and Woodson 2008; Updegrove and Wartell 2011). In a functional context, Hfq is known to enhance the interaction between RprA and *rpoS* (Updegrove et al. 2008). RprA is predicted to contain multiple stem–loops that are thought to block the required site for pairing to *rpoS* (Fig. 1A; Soper et al. 2010). Therefore, the large RprA structural changes observed upon interaction with Hfq suggests Hfq alters the structure of the RprA to facilitate its pairing to *rpoS*. RprA itself is proposed to straddle the distal face of Hfq, but the ab initio model indicates that significant proportions of the distal and lateral surfaces remain exposed. This suggests that the RprA:Hfq hexamer complex could potentially accommodate *rpoS* binding to the exposed regions of the distal and lateral surfaces of Hfq, thereby forming a ternary complex. This raises the possibility that the close proximity of *rpoS* and RprA binding to the distal face of Hfq could aid RprA:*rpoS* pairing. For OxyS, the role of Hfq in enhancing the interaction between OxyS and *rpoS* is less understood (Updegrove and Wartell 2011). Nevertheless, the OxyS:Hfq hexamer ab initio model similarly has exposed regions within the distal and lateral surfaces available for *rpoS* binding. With OxyS proposed to partially contact the distal face, pairing to distal face-bound *rpoS* could similarly be supported. Likewise, *fhlA*, another down-regulated mRNA target of OxyS, also has ARN repeats and has been shown to bind to the distal face of Hfq (Salim and Feig 2010). Consequently, with exposed distal face regions within the OxyS:Hfq hexamer complex available for *fhlA* binding, pairing between distal face–bound OxyS and *fhlA* could similarly be supported (Altuvia et al. 1998).

In this work we identify another role for Hfq in enhancing the RNase E–mediated degradation of OxyS. Hfq and RNase E share similar RNA binding/cleavage site preferences (AU-rich regions adjacent to a stem–loop) (de Haseth and Uhlenbeck 1980; McDowall et al. 1994, 1995; Moll et al. 2003; Prevost et al. 2011), and a level of RNase E cleavage of OxyS is observed in the absence of Hfq, indicating such a site to exist in OxyS. However, while Hfq may also bind to this shared Hfq binding/RNase E cleavage site, it fails to block RNase E cleavage and instead enhances degradation. We propose that the OxyS structural changes induced by Hfq create a more preferable RNase E cleavage site, such that degradation of OxyS is enhanced. Recent studies involving MicC, an sRNA that pairs to its mRNA target in the coding region and functions in its down-regulation, indicate that the role of processed MicC (containing a 5′ monophosphate) is to guide and activate RNase E to cleave its paired target mRNA leading to mRNA signal removal (Bandyra et al. 2012). However, if processed MicC fails to pair to its target mRNA, MicC enters a discard pathway in which it is rapidly degraded by RNase E in the presence of Hfq (Bandyra et al. 2012). Like MicC, OxyS is also involved in down-regulating its mRNA target, *rpoS*. Hence, the enhanced RNase E–mediated degradation of processed OxyS bound to Hfq could be illustrative of the discard pathway, suggesting that Hfq may have a role in regulating OxyS signal removal and thereby control the levels of OxyS available to function. Considering the role of OxyS in down-regulating *rpoS*, it could function similarly to MicC in terms of the processed OxyS guiding and activating RNase E to cleave the paired mRNA target. However, unlike MicC, which pairs to its mRNA target in the coding region (Pfeiffer et al. 2009; Bandyra et al. 2012), OxyS has been proposed to pair to the RBS of its mRNA target, *rpoS* (Zhang et al. 2002), although, interestingly, OxyS has previously been shown to pair to *fhlA*, another mRNA target within both the RBS and the coding region (Argaman and Altuvia 2000). With these differing mRNA-target pairing sites, whether, like MicC, OxyS can also target paired *rpoS* or *fhlA* for RNase E degradation, currently remains unknown.

In contrast to the situation for OxyS and MicC (Bandyra et al. 2012), Hfq provides a protective effect on RprA in the presence of RNase E. With the similar sRNA binding preferences of RNase E and Hfq, it is probable that Hfq simply blocks the site that is also recognized by RNase E, resulting in a decrease in RprA degradation. Alternatively, the Hfq-induced structural change in RprA may remove the RNase E cleavage site altogether, similarly providing an RprA stabilization effect. Unlike OxyS, RprA is involved in up-regulating expression of the common mRNA target, *rpoS*, and it appears that as processed RprA is not targeted for RNase E degradation, the discard pathway (Bandyra et al. 2012) potentially identified for OxyS does not apply to RprA. Considering whether this RprA protection from RNase E degradation upon Hfq binding is transmitted to *rpoS*, within the context of a ternary complex, earlier in vivo studies indicate that *rpoS* is degraded via an RNase E–mediated mechanism but is stabilized in the presence of RprA (McCullen et al. 2010). Together these data suggest that the protective effect of Hfq on RprA is likely transmitted to its paired mRNA target, *rpoS.* Additionally, RprA is proposed to bind to the distal face of Hfq, and previous findings suggest that sRNA distal-face binders, such as MicM (which contains four ARN repeats), demonstrate protection to RNase degradation, possibly for the purposes of sRNA recycling (Olsen et al. 2010). Protecting RprA from RNase E degradation may well therefore support its repeated use in the cell, possibly within the context of sRNAs cycling on Hfq (Fender et al. 2010; Sauer et al. 2012). While MicM and potentially RprA are both distal-face binders, MicM is involved in mRNA-target down-regulation through pairing to the RBS of its mRNA target (Rasmussen et al. 2009). In contrast, RprA is involved in up-regulation of its mRNA target, *rpoS*, through pairing to the 5′ UTR of *rpoS*, relieving the inhibitory secondary structure around the RBS and thereby supporting its translation (Updegrove et al. 2008). Interestingly, RprA has recently been identified as being involved in down-regulation of two other mRNAs, namely, *csgD* and *ydaM* (Mika et al. 2012). Hence while RprA protection from RNase E appears to be transmitted to its mRNA target, *rpoS*, to support up-regulation, the full picture is evidently complex with no immediate correlation between RNase E protection and sRNA function.

Collectively, our data show that Hfq can interact with, and change the structures of, RprA and OxyS, without significantly altering its own conformation. This illustrates the flexibility of Hfq’s role in supporting sRNA-mediated mechanisms. In most cases, Hfq has been shown to be involved in enhancing sRNA–mRNA pairing (Soper and Woodson 2008; Updegrove et al. 2008). However, our findings suggest that while Hfq is important in mediating sRNA structure changes, potentially as a means of enhancing pairing to partner mRNAs, this may not always be Hfq’s primary role. We demonstrate that the sRNA structure changes induced by Hfq binding can impact sRNA stability to RNase E degradation, highlighting it to have an important role linked to the control of functional sRNA levels. Additionally, while the low-resolution structural information presented here has provided key insights, the nature of the sRNA structural changes induced by Hfq remain to be elucidated, as do the structural details within the context of the mRNA-containing ternary complex. It is only with such information that a detailed knowledge of how the opposing regulatory functions of RprA and OxyS, expressed in response to differing stress conditions, are communicated to their common mRNA target, *rpoS*, via the action of Hfq will be obtained.

## MATERIALS AND METHODS

### Protein expression and purification

Plasmid containing the gene encoding *E. coli* Hfq [pEH-10(hfq)] was the kind gift of I. Moll (Max F. Perutz Laboratories, University of Vienna, Austria). BL21 (DE3) cells transformed with pEH-10(hfq) were grown at 37°C in LB medium supplemented with 100 μg/μL ampicillin to an OD_600_ of 0.6. Protein expression was induced with 1 mM IPTG, and the cells were left to incubate for 3 h before harvesting by centrifugation (4000 rpm, 20 min, 4°C). Hfq was purified according to the method described previously (Vassilieva et al. 2002; Henderson et al. 2013). All Hfq concentrations used relate to the protein in its hexameric form.

Plasmid containing the gene encoding *E. coli* RNase E 1-529, catalytic domain with an N-terminal oligo His-tag (pRne 529-N) was the kind gift of Ben Luisi (Cambridge University, Cambridge, UK). BL21 (DE3) cells transformed with pRne 529-N were grown, at 37°C in LB medium supplemented with 100 μg/μL ampicillin, to an OD_600_ of 0.6. Protein expression was induced with 1 mM IPTG, and the cells were left to incubate for 3 h before harvesting by centrifugation (4000 rpm, 20 min, 4°C). RNase E was purified according to the method described previously (Callaghan et al. 2003). All RNase E concentrations used relate to the protein in its tetrameric form.

### Preparation of RNAs

DNA templates encoding OxyS and RprA were generated through the extension of overlapping primers (Gao et al. 2003) with KOD Hot start polymerase. For the primer sequences, see Table 1. Each sequence was designed to contain a T7 promoter sequence (5′-TAATACGACTCACTATA) and up to three guanines at the 5′-end to enhance the yield from transcription. Analysis by Mfold (Zuker 2003) indicated that these additional guanines would not be expected to affect the RNA structure. RNAs were transcribed in vitro by T7 RNA polymerase (Ambion Megascript kit) over 4 h. Template DNA was removed with TurboDNase and the remaining RNA purified (Ambion MegaClear kit). To produce radiolabeled RNAs, [^32^P]pCp (cytidine bis-phosphate) was attached to the 3′ terminal using RNA ligase. To produce monophosphorylated RNAs, the 5′ triphosphate was first removed with Antarctic phosphatase (NEB) before a single phosphate was replaced using polynucleotide kinase. All RNAs were confirmed to be of a high purity by gel analysis (data not shown). Prior to use, all RNAs were denatured for 2 min at 80°C and then cooled for 5 min at room temperature to allow them to refold in their experimental buffer. RprA has a theoretical molecular mass of 35,077 Da, and OxyS has a theoretical molecular mass of 36,426 Da.

**TABLE 1.**
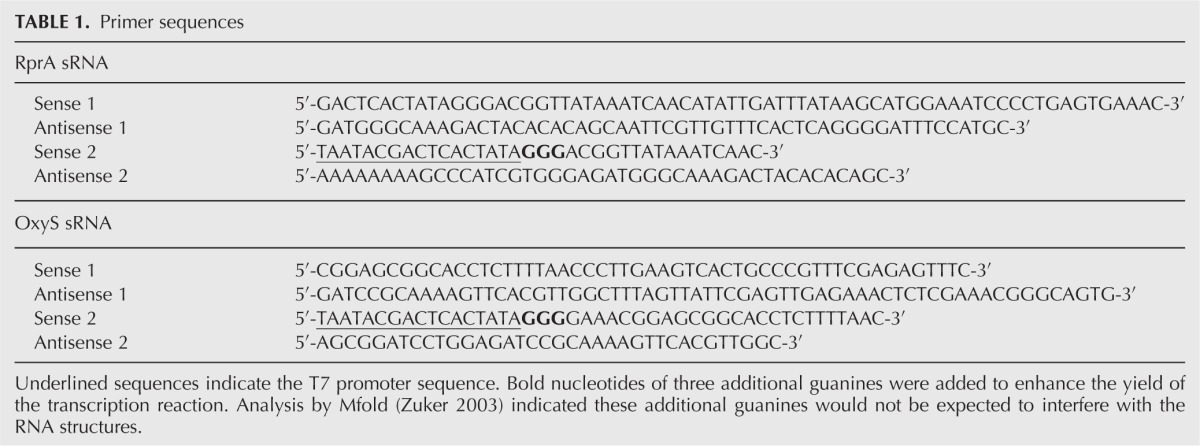
Primer sequences

### Nondenaturing MS

Nondenaturing MS was performed using equimolar solutions of sRNA (OxyS or RprA) and Hfq in 250 mM ammonium acetate (pH 7). Samples were injected using nano-electrospray ionization (Advion Triversa Nanomate; spray voltage 1.75 kV) into a quadrupole-time of flight mass spectrometer (Waters Synapt GI) under conditions suitable for the preservation of noncovalent interactions. Key settings for OxyS:Hfq were as follows: ion mobility mode; sampling cone, 190 V; extraction cone, 5 V; trap collision energy, 75 V; transfer collision energy, 15 V; and trap DC bias, 30 V; and trap, 5 V. For RprA:Hfq, the key settings were as above with the exception of the following: trap collision energy, 90 V; transfer collision energy, 12 V; bias, 30 V; and trap, 5 V. Data were processed and smoothed using a Savitsky-Golay algorithm with ±50 channels wide using MassLynx software, version 4.0.

### Analytical ultracentrifugation

Sedimentation velocity was carried out in a Beckman Optima XLA analytical ultracentrifuge using an AnTi-50 8 hole rotor. Four hundred microliters of 800–900 nM 1:2 sRNA:Hfq hexamer (complex II) in 10 mM Tris-HCl (pH 8.0), 50 mM NaCl, and 50 mM KCl was loaded into a double sector cell (12-mm path length) with 425 μL in the reference channel. The samples were sedimented at 30,000 rpm at 10°C with radial absorbance scans measured every 10 min at 265 nm. Data were fitted with SEDFIT (Schuck 2000).

### Native EMSA

RNAs were heated for 2 min to 80°C and then cooled for 5 min at room temperature to allow them to fold. All binding assays were carried out in 10 µL volumes, unless otherwise stated, in the following buffer: 10 mM Tris-HCl (pH 8.0), 50 mM NaCl, 50 mM KCl, and 10% glycerol; lacking in Mg^2+^ to ensure consistency with the conditions used for earlier EMSA studies (Updegrove et al. 2008; Soper et al. 2010; Updegrove and Wartell 2011), although we note that Mg^2+^ failed to impact the interactions seen (data not shown). Reactions were electrophoresed on 6% native PAGE run in 90 mM Tris-Borate, 1 mM EDTA (TBE) at 100 V for 1.5 h. Gels were dried, imaged with a Fujifilm phosphorimager (FLA-5000), and analyzed with MultiGauge software. For Hfq–RNA interactions, the fraction of ^32^P-labeled RNA in each sRNA complex was calculated as a proportion of the total counts in each lane and fit either to a partition function for cooperative binding of two independent sites, according to the method described previously (Lease and Woodson 2004), or to a quadratic binding function. In these experiments, 0–100 nM concentrations of Hfq hexamer were used. Previous studies suggest that Hfq would be monomeric at these concentrations (Panja and Woodson 2012); however, we demonstrate Hfq to be in its hexameric form in our preparations (Henderson et al. 2013).

### Surface plasmon resonance

Hfq hexamer was injected over an ethyl(dimethylaminopropyl) carbodiimide (EDC)/*N*-hydroxysuccinimide (NHS)–activated CM5 chip at 10 µL/min until 100–500 arbitrary response units (RUs) of sample were immobilized by amine-coupling. To monitor binding interactions with the sRNAs, single-cycle kinetics experiments were performed. This involved five consecutive injections of increasing concentrations of sRNAs (0–50 nM RprA or 0–25 nM OxyS sRNAs). sRNAs were flowed at 10 µL/min in 10 mM HEPES, 150 mM sodium chloride, 3 mM EDTA, and 0.005% Tween20. The buffer lacked Mg^2+^ to ensure consistency with the conditions used previously for Hfq–sRNA interaction studies (Updegrove et al. 2008; Soper et al. 2010; Updegrove and Wartell 2011). Data were analyzed using Biacore BiaEvaluation software and fitted with a 1:1 binding model.

### Circular dichroism

CD experiments were performed on an Applied Photophysics π*-180 spectrometer at 20°C. RprA and OxyS were buffer-exchanged into 10 mM Tris-HCl (pH 8.0) and 100 mM NaCl, denatured for 2 min to 80°C, and cooled for 5 min at room temperature to allow them to refold. Mg^2+^ was omitted from the buffer in order to maintain consistency with the conditions used in earlier sRNA–Hfq interaction studies (Updegrove et al. 2008; Soper et al. 2010; Updegrove and Wartell 2011), and we note that RprA was demonstrated to have the same structure ± Mg^2+^ (data not shown). RNAs (1 μM) were mixed with 0 μM, 1 μM, and 2μM Hfq hexamer and measured in a 0.4-mm path-length cuvette over a wavelength range spanning 200–350 nm in 1-nm step sizes. The protein contribution was subtracted, and four to six scans were averaged. The baseline was subtracted and data smoothed using the Savitsky-Golay routine to reduce noise. The spectra were converted into molar ellipticity units (deg.cm^2^/dmol).

### Small-angle X-ray scattering

SAXS experiments were performed on the ID14-3 bioSAXS beamline at the European synchrotron radiation facility (ESRF) with a wavelength of 0.931 Å and a camera length of 2.42 m, covering a q range of 0.005–0.5 Å^−1^ (where q is the scattering vector [4πsinθ/λ]).

Using Amicon Ultra 0.5 mL 10 kDa centrifugal concentrators, Hfq, OxyS, RprA, and tRNA (as a calibration control) were buffer exchanged in to 10mM Tris-HCl (pH 8.0), 50 mM NaCl, and 50 mM KCl. The buffer used lacked Mg^2+^ to ensure consistency with the conditions used for earlier sRNA–Hfq interaction and SAXS studies (Updegrove et al. 2008; Soper et al. 2010; Updegrove and Wartell 2011; Ribeiro Ede et al. 2012; Vincent et al. 2012b). Data were collected at 25°C for three concentrations of each sample; OxyS (10, 22.3, and 39 μM), RprA (8.9, 19.9, and 33 μM), Hfq hexamer (29.5, 75, and 149 μM), 1:1 OxyS:Hfq hexamer (8.8, 18.8, and 37.8 μM), 1:2 OxyS:Hfq hexamer (6.2, 17.8, and 27.4 μM), 1:1 RprA:Hfq hexamer (9.5, 19, and 35.5 μM), and 1:2 RprA:Hfq hexamer (8.4, 12.2, and 26.3 μM). Ten × 10-second frames were acquired under a constant flow rate to avoid the effects of radiation damage. Similarly, data were collected in the same manner for each of the 1:1 sRNA:Hfq complexes prepared for SANS analysis (see below).

Scattering curves were buffer subtracted and merged using Primus software (Konarev et al. 2003). At low angles, the *R*_g_ was found using the Guinier approximation, *I*(q) = *I*(0) exp ^1^/_3_*R*_g_^2^q^2^, with *I*(0) indicating forward scattering intensity. Transformation of the scattering curve by the GNOM program (Svergun 1992) generated a distribution of particle distances, allowing the maximum dimension to be determined, *D*_max_. Conformation of correct dimensions was achieved when the *R*_g_ from GNOM matched that obtained from the Guinier approximation. *DAMMIF* was used to make low-resolution ab initio models of the SAXS data (Franke and Svergun 2009). Twenty models were generated, averaged by Damaver, and filtered with Damfilt to make a model that represented the most probable averaged conformation (Volkov and Svergun 2003). The molecular masses of the sRNA:Hfq complexes were obtained using the equation below, while *I*(0) values were corrected using tRNA as a secondary standard. 

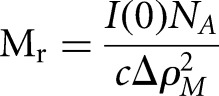

 where *c* is the concentration of the sample (g.cm^−3^), *N*_A_ is Avogadro’s number, and Δρ_*M*_^2^ is the square of the scattering contrast per unit mass. Δρ_*M*_^2^ is calculated from the equation Δρ_*M*_^2^ = (Δρ*v*)^2^, where Δ*ρ* is the contrast or difference in scattering density between the solvent and the scattering particle, and *υ* is its partial specific volume (cm^3^/g). The program MULCh (ModULes for the analysis of contrast variation data) (Whitten et al. 2008), was used to calculate the theoretical value of the scattering length density and scattering length difference, based on the amino acid sequence for the protein and/or nucleotide sequence for the sRNA substrates and buffer composition.

### Small-angle neutron scattering

SANS experiments were performed at the Institut Laue-Langevin (ILL), Grenoble, on the D22 beamline. Measurements were recorded with a 2-m and 11.2-m detector distance covering a q range of 0.01–0.3 Å^−1^ at a wavelength of 6 Å. RprA and OxyS sRNAs were denatured for 2 min at 80°C followed by 5 min at room temperature to allow them to refold. Both were mixed with Hfq hexamer and the complexes buffer-exchanged into Tris-HCl/Tris-DCl (pH 8.0), 50 mM KCl, and 50 mM NaCl, with 0%, 20%, 40%, 60%, or 73% D_2_O. The 1:1 sRNA:Hfq hexamer complexes formed were confirmed to be homogeneous by native gel analyses. Sample concentrations were between 3–4 mg/mL of complex. Data were reduced, and buffer, noise, and intensity were corrected using GRASP V5.09. Data were merged from both detector distances and analyzed as per the SAXS data to generate the *R*_g_ and *D*_max_ values.

Ab initio modeling of OxyS:Hfq and RprA:Hfq was performed using the multiphase dummy-atom modeling program MONSA (Svergun and Nierhaus 2000). This program attempts to minimize the discrepancy between the fit of the model and the experimental data and describes the model as an assembly of beads within a spherical search volume with the diameter equal to that of the complex. The protein phase (Hfq) was first modeled using DAMMIF (Franke and Svergun 2009) with P6 symmetry imposed. The model was centered on the origin with MASSHA (Konarev et al. 2001), and a spherical search volume of either 145 Å for RprA:Hfq or 155 Å for OxyS:Hfq was created (on the basis of the *D*_max_ value determined from the *P*(*r*) (pair distribution function) of the SAXS data) using the auxiliary ATSAS program “pdb2dam4” (kind gift from Maxim Petoukhov). The following edits were made to the coordinates output to maintain the Hfq phase during MONSA: “H_space_” was replaced with “CA,” “0 1 201” was replaced with “0 1 202,” and “0 2 201” was replaced with “1 2 201.” These files were used as the starting search volume in MONSA and effectively “fixed” the protein phase of the complex.

In the case of OxyS:Hfq, three data sets (0%, 20%, and 60% D_2_O SANS data) were used during the modeling process to determine the conformationally averaged structure of OxyS bound to Hfq, where the 0% D_2_O SANS data represents the scattering from the whole of the complex and the 20% and 60% D_2_O SANS data represent the scattering either side of the contrast match point for the sRNA component. In the case of RprA:Hfq, two data sets were used: the 0% SANS data representing the scattering for the whole complex and the 40% D_2_O SANS data representing the scattering primarily due to RprA in complex with Hfq. The second (RNA) phase left for MONSA to determine was either the OxyS or RprA in complex with Hfq. No symmetry was imposed for the model of the whole complex. Theoretical volumes based on the amino acid or nucleic acid sequence and *R*_g_ values determined from Guinier analysis were used as further constraints during the modeling process. The contrast was calculated using the program MULCh (Whitten et al. 2008).

### RNase E cleavage assay

RNA in the presence and absence of Hfq was incubated with purified recombinant *E. coli* RNase E over 15 min at 37°C in 10 mM Tris-HCl (pH 8.0), 50 mM NaCl, 50 mM KCl, 10 mM MgCl_2_, and 10% v/v glycerol in 10 µL volumes. Aliquots were removed at set time-points and quenched with 50 mM EDTA to stop the reaction. One microliter of proteinase K and 6.25% SDS was added and the samples incubated for 10 min at 50°C. The RNAs were then ethanol precipitated using 1/10 volume of 3M sodium acetate (pH 7) and 3 volumes chilled 100% ethanol. These were incubated for 2 h at −80°C and centrifuged at 17,000*g* for 30 min. The resulting pellet was washed in 800 μL fresh 70% ethanol and recentrifuged for a further 20 min at 17,000*g*. The pellet was dried, resuspended in 5 μL loading dye, heated for 5 min at 95°C, and loaded onto a 6% denaturing PAGE gel. The gels were run in 1× TBE at 200 V at room temperature before staining with SYBR Gold. Gels were imaged with a UV transilluminator and the quantity of intact sRNA determined with MultiGauge software. GraFIT5 (Erithacus Software) was used to identify the initial rate of degradation over the first 3 min of the reaction (the linear phase).
